# Some Clinical Observations on the Prophilaxis Treatment of Uric Acid Conditions

**Published:** 1904-06

**Authors:** 


### SOME CLINICAL OBSERVATIONS ON THE PROPHILAXIS TREATMENT OF URIC ACID CONDITIONS.*

The importance of promptly recognizing the immediate and remote effects of the baneful influence of uric acid excess on the blood and tissues of the organisms and the good results from the proper management of such conditions, contrasted with the unsatisfactory results frequently found in their treatment is my apology for addressing you upon this well worn and hackneyed subject.

How much of function and of life might be restored and maintained by the intelligent management of such conditions! How much suffering, heart disease, chronic nephritis, arteric scelrosis and many other similar conditions averted by neutralizing the effects of this irritant upon the tissues. Statistics show that about one-third of all cases of heart disease are due to rheumatism. I believe that a great many cases of chronic nephritis, and my experience corroborates it, are undoubtedly due to the irritating

*Presented by J. C. Walton, M.D., to the Tri-State Medicai Association of the Carolinas and Virginia at the Danville, meeting February, 1904.

effects of uric acid upon the uriniferous tubules. I have no new theories to exploit, but having had rather unusual facilities and considerable experience along these lines in sanitorium work, where the patients were kept under close observation and were enabled to get the benefit of modern methods of treatment, especially as regards diet, regime, etc., I will as briefly as possible give the line of treatment found most satisfactory in such conditions, and can commend the same. The cases treated included many of gout, rheumatism, myalgia, sciatica, lumbago, neuralgia, chronic nephritis, arteric sclerosis and gastro-intestinal troubles. A careful urinary analysis was made in nearly all the cases and almost invaribly a large excess of uric acid was found, which gradually subsided and approached the normal as the disease yielded to treatment. A noticeable fact was that the more chronic the conditions the less amenable it was to anything like drug medication and the nearer we approached the lines of physic therapy, or nature's own methods, the more satisfactory were our results, including such matters as diet, exercise, clothing, water drinking, bathing, hot air, electricity, massage, etc.

*Diet* is of vital importance. No matter how thorough and complete elimination may be, you have got to limit the uric acid intake while trying to increase the output to the utmost. Avoid red meats, pork and beef, tea, coffee, cocoa, tomatoes, rhubarb and an excess of sweets and starches. Allow fruits, vegetables, mutton, fowl, fish, oysters, cheese, butter and milk. Whether tea and coffee, according to Haig, contain large quantities of uric acid or their equivalent Xanthin compounds, I am not prepared to say, but I believe that they are especially unsuited for rheumatism. One case that I recall, who after returning from Hot Springs, Ark., and his rheumatism persisting notwithstanding a rigid diet otherwise, with the single exception of his coffee, and after stopping the coffee the disease promptly subsided. I have been impressed with this fact sufficiently often in other cases to convince me of the bad effects of coffee, tea and chocolate under such conditions.

*Exercise*, especially in the open air. Human beings, like plants, require an abundance of fresh air and sunshine. Keep the skin, bowels, kidneys and all of the eliminating organs at work. Lack of exercise, over-eating, drinking too small quantities of water, and the consequent retention of toxic substances in the blood, non-elimination, autointoxications are sources of the most potent causes of disease. Get a hump on yourself; step lively and sweat a little and keep your sewers open. There is one thing a rheumatic should never do, i. e., giving up; keep going. The worst case of anthritis deformans I ever saw, ankylosed joints, bad heart, pronounced hopeless by the best physicians, got for a time relief from his pain with hot air and keeps on the road constantly and says that the only thing that has kept him alive is his full determination not to give up the ship. In regard to auto intoxicants: In a recent conversation, a noted New York surgeon who is celebrated for his hard horse sense remarked, that the most valuable lesson of a busy life was the importance of recognizing auto intoxication, especially of the gastro intestinal tract as the majority of mankind are poisoned by their own toxines.

*Clothing.*—These subjects are very susceptible to sudden temperature changes and should wear flannels the year round; light woolen, of course, for warm weather, as the heavy ones are too depressing.

*Water Drinking.*—It is surprising how few people drink enough water—water being the natural sewer of the body, the great solvent and eliminator, flushing out all the channels of the body, eliminating and dissolving the toxines, keeping the secretions going and promoting a general feeling of well-being. Drink from one to four quarts daily. Don't drink during or after meals, as it interferes with digestion by diluting the gastric juice. Always drink pure water, preferably a good alkaline mineral water containing a good per cent of potash and soda, as they are among the best acid solvents.

*Bathing.*—The skin is by far the best medium for the elimination of uric acid, and other noxious products, and they can be more rapidly and safely eliminated this way than any other. The skin is rather a lazy, sluggish organ; the pores are easily clogged up and it frequently requires a lot of stimulation by friction, heat, etc., whipping up, you might say, before it can be made to do its duty and perform its functions. One of the worst cases of gout I ever tackled was a man of 30 ; came on in infancy. He had been to Hot Springs three times, with very little benefit. At the beginning of treatment it required 40 minutes in a hot air cabinet at a temperature of 165 degrees to produce a good sweat; later as improvement progressed, the perspiration came on in shorter time, and finally in 20 minutes and as the action of the skin approached the normal, the disease subsided and convalescence was rapid. In private practice hot baths are most convenient, but in institution work the Baruch tonic bath is far and away ahead of any system of baths known to the medical profession. By means of the hot air cabinets we can regulate the perspiration and get all of the elimination needed, and this can be followed by a system of graduated douches which tone up the nervous and vascular systems acting as a powerful reconstructive and tonic and prevent the taking of colds, that bug-bear of that abomination, the Turkish and Russian bath.

Dry hot, or super-heated air : One of the most rapid eliminators of toxines known, so much so that Hare in his system of therapeutics states that there is sometimes danger of lighting up an acute exacerbation of gout or rheumatism from too suddenly throwing into the circulation large quantities of uric acid. This method of treatment is admirably adapted to all painful and inflammatory conditions, especially sprains, luxations, chronic and subacute articular rheumatism, synovitis, ankylosis, adhesions, exudations, deposits, etc. It is astonishing how old deposits and adhesions, unless organic in nature, can be dissolved and broken up, especially when followed by judicious massage.

Electricity, especially static. I know of no agent as effective for the relief of all painful conditions whether of joints, nerves or muscles, and as a general all round tonic, sedative or invigorator, than static electricity. Is simply invaluable, old sciatias, lumbagos, the pains of locomoter ataxia and general neuralgic condition, frequently yield to static electricity, after all known measures have failed. As a sedative, as a hypnotic and in all neurasthenic conditions, it is unrivalled. Finally, an invaluable adjunct to all these is a good masseur. A good one is hard to find, but they are simply indispensable.

In conclusion, gentlemen, the short time at my disposal, does not allow me to even approximate justice to this important subject, and the most I could hope for is to call your attention briefly to a few of the important landmarks in the prophylaxis and treatment of such conditions.

### A BRIEF REPORT OF CASES.

Mr. H., aged 30, inveterate case of sciatica, right side of over a year's duration, complicated with subacute articular rheumatism of the left knee joint. Usual treatment, Baruch baths, electricity, etc. Case very rebellious, and only finally yielded to bombardment of the nerve with heavy static sparks daily. Dismissed case cured after four weeks' treatment. In this case is strikingly illustrated the value of electricity.

Mr. W., prominent attorney, ill more than a year. Case pronounced rheumotoid arthritis, and prognosis hopeless. Had spent some time in hospitals in Hot Springs, Va., and had exhausted all of the usual orthodox treatment with no benefit. General condition very bad; required large doses of opiates at night and rested very badly. Both shoulder joints very stiff and painful, could not use arms or hands. After trying the usual treatments with no benefit whatever, and as a dernier resort, put him upon hot air and massage, using the hot air for one hour daily, followed by thorough massage, with gradual relief and complete recovery in a few weeks. Has remained so. This case strikingly shows the benefit of hot air and massage, and I don't believe anything else would have cured him.

Young man was affected with attacks of rheumatic gout from infancy. Had been to Hot Springs, Ark., three times. Disease confined to the feet and knees. Baruch baths and electricity effected a cure in a few weeks. Relief from the baths in this case was striking. Another case, skiagraphs, showed a calculi in each ureter. Patient suffering considerably with pains over the kidneys and ureter. Two weeks' treatment with Baruch baths and static electricity, entirely relieved pain and apparently produced a cure. Patient left suddenly and unfortunately was unable to get another picture. Was well when heard from several weeks later.—*Carolina Medical Journal.*

